# The Use of Iodide of Potassium and Calomel in Diseases of the Eye

**Published:** 1880-03

**Authors:** Lucien Howe


### TRANSLATIONS.

---

### THE USE OF IODIDE OF POTASSIUM AND CALOMEL IN DISEASES OF THE EYE.

FROM THE GERMAN, BY LUCIEN HOWE, M. D.

TWELVE years ago Hennequin called attention to an acute inflammation of the eye, produced by the internal use of iodide of potassium, in connection with the dusting of calomel upon the conjunctiva. This effect was not then entirely unknown, having already been discovered thirty years before by Fricke, and verified by others, (Fretschi, Andreal and Edm. Rose) the inflammation being attributed to the peculiarly irritating action of iodide of mercury, which, according to chemists, must be formed on the conjunctiva under such circumstances. The author, by means of certain experiments on animals, and by chemical analysis, has considered the question carefully, and arrived at the important practical conclusion, that, the external use of calomel is to be avoided, whenever iodine is contained in the tears. For, when iodide of potassium is administered internally, it can in a short time—even in a few moments—be detected in the different secretions and excretions, and especially in the tears.

A dose of 0.50 centigrams, (grs. viii.) taken twice in twenty-four hours, is sufficient to keep it continually present in the tears of the human subject.

Now, although calomel is but slightly soluble in water, alone, it is much more readily dissolved in a solution of ¾ per cent. of common salt, and can consequently always be taken up by the fluids on the conjunctiva. Therefore, when calomel is dusted on to the membrane, simultaneously with the administration of the iodide of potassium internally; iodides are formed which act as irritants to the conjunctiva, producing an acute inflammatory condition. (Archiv für Ophthalmologie—Klinische Monatsblätter für Augenheilkunde.)

[Note. Reference has already been made to this subject, by the writer, in a former number of the BUFFALO MEDICAL AND SURGICAL JOURNAL, vol. xv, p. 145.]

In a large class of cases—especially where phlyctenulæ appear on the conjunctiva or cornea—it has been customary to apply calomel in the manner indicated, and at the same time to advise the internal use of some form of the iodides. Only recently, however, have the details of the process been carefully studied and the action of the two agents fully demonstrated by exact chemical investigation. The evil effect of such a procedure, is however thus proved by chemical analysis, as it was before shown by clinical experience.

